# Natural immune response to *Plasmodium vivax* alpha-helical coiled coil protein motifs and its association with the risk of *P*. *vivax* malaria

**DOI:** 10.1371/journal.pone.0179863

**Published:** 2017-06-26

**Authors:** Nora Céspedes, Connie S. N. Li Wai Suen, Cristian Koepfli, Camila T. França, Ingrid Felger, Issa Nebie, Myriam Arévalo-Herrera, Ivo Mueller, Giampietro Corradin, Sócrates Herrera

**Affiliations:** 1 Malaria Vaccine and Drug Development Center (MVDC), Cali, Colombia; 2 School of Health, University of Valle, Cali, Colombia; 3 Population Health & Immunity Division, Walter and Eliza Hall Institute of Medical Research, Parkville, Australia; 4 Department of Medical Biology, University of Melbourne, Melbourne, Australia; 5 Swiss Tropical and Public Health Institute, Basel, Switzerland; 6 Centre National de Recherche et de Formation sur le Paludisme, Ouagadougou, Burkina Faso; 7 Malaria: Parasites and Hosts Unit, Department of Parasites & Insect Vectors, Institut Pasteur, Paris, France; 8 Barcelona Institute of Global Health (ISGLOBAL), Barcelona, Spain; 9 Biochemistry Department, University of Lausanne, Epalinges, Switzerland; 10 Caucaseco Scientific Research Center, Cali, Colombia; Universidade Federal de Minas Gerais, BRAZIL

## Abstract

Protein α-helical coiled coil structures are known to induce antibodies able to block critical functions in different pathogens. In a previous study, a total of 50 proteins of *Plasmodium vivax* erythrocytic asexual stages containing α-helical coiled coil structural motifs were identified *in silico*, and the corresponding peptides were chemically synthesized. A total of 43 peptides were recognized by naturally acquired antibodies in plasma samples from both Papua New Guinea (PNG) and Colombian adult donors. In this study, the association between IgG antibodies to these peptides and clinical immunity was further explored by measuring total IgG antibody levels to 24 peptides in baseline samples from a longitudinal study of children aged 1–3 years (n = 164) followed for 16 months. Samples were reactive to all peptides tested. Eight peptides were recognized by >50% of individuals, whereas only one peptide had < 20% reactivity. Children infected at baseline were seropositive to 23/24 peptides. No significant association was observed between antibody titers and age or molecular force of infection, suggesting that antibody levels had already reached an equilibrium. There was a strong association between antibody levels to all peptides and protection against *P*. *vivax* clinical episodes during the 16 months follow-up. These results suggest that the selected coiled coil antigens might be good markers of both exposure and acquired immunity to *P*. *vivax* malaria, and further preclinical investigation should be performed to determine their potential as *P*. *vivax* vaccine antigens.

## Introduction

*Plasmodium vivax* is the second most important *Plasmodium* species in terms of epidemiological significance, with an estimate of 13.8 million malaria cases globally in 2015, and about half of the total number of malaria cases occurring outside Africa [1]. Although this parasite has been classically considered benign, several features make it difficult to control and eliminate. First, severe and lethal *P*. *vivax* malaria cases have been reported [1–4]; second, chloroquine-resistant strains have recently emerged with at least one case confirmed in 10 countries [1]; third, this parasite species produces hypnozoite-forms that upon periodic reactivation induce clinical relapses [5, 6], even in individuals who have left endemic regions; and fourth, gametocytes emerge early during the erythrocytic cycle possibly increasing its transmissibility [7, 8]. Because of the difficulty in controlling *P*. *vivax*, in areas where the two species coexist its incidence appears to decrease more slowly than that of *Plasmodium falciparum* [1].

Due to limitations associated with classical malaria control measures, vaccination against malaria is currently considered a potentially valuable cost-effective complement for malaria control activities that would significantly contribute to its elimination [9]. During the last 2–3 decades, significant efforts have been invested on developing *P*. *falciparum* [10], and more recently *P*. *vivax* vaccines [11]. However, discovery of new potential vaccine candidates is required. The use of bioinformatics tools has allowed to explore the malaria genome/proteome databases, and to identify parasite proteins containing specific domains with functional importance for the parasite that could be immunologically targeted and therefore represent novel candidate antigens for vaccine development.

Protein α-helical coiled coils are stable structures capable of eliciting antibodies able to block functional domains in different microorganisms [12–14]. These motifs have been investigated in influenza virus [13, 15], HIV-1 [12], coronaviruses [14] and malaria parasites [16]. In the case of *P*. *falciparum*, 170 α-helical coiled coil motifs have been identified *in silico*, from proteins predicted to be in different cellular locations such as the cytoplasm, the nucleus, the mitochondria, and the peroxysomes and in addition, some of them have trans- membrane segments. Therefore, synthetic peptides containing these motifs were synthesized, and tested for their reactivity in serum obtained from adult donors from Burkina Faso, Tanzania and Colombia [17]. The most recognized antigens were selected, and specific human IgG antibodies were affinity purified and tested *in vitro* using antibody-dependent inhibition (ADCI) assays, showing that several of them were active in inhibiting *in vitro* parasite growth [17, 18]. Association of antibody responses with protection against infection was also observed [18, 19]. In the case of *P*. *vivax*, 43 coiled coils segments with extensive homology to the *P*. *falciparum* counterparts were identified *in silico*, and the synthesized segments were tested for their reactivity with serum of individuals from malaria endemic areas [20]. However, due to the lack of *P*. *vivax in vitro* cultures, the functional activity of antibodies elicited to these antigens has not yet been studied in this parasite species. Similarly, there are no reports on the relationship between antibody responses to *P*. *vivax* coiled coil antigens and risk of infection or clinical malaria.

In order to explore the association between total IgG antibodies to *P*. *vivax* coiled coil segments (selected based on their antigenicity) and risk of disease, baseline plasma samples from a cohort of children from Papua New Guinea (PNG) [21] were tested using a multiplexed bead array assay. In this cohort, 264 children aged 1–3 years were enrolled and actively followed for up to 16 months to identify factors associated with either risk of or protection from infection and disease, and the molecular force of blood-stage infection (_mol_FOB) at the individual level was also assessed [22, 23]. Exposure to *P*. *vivax* infections varied greatly (between 0 and 38 clones acquired over the entire study period), and children were found to acquire an average of 15 new *P*. *vivax* blood-stage clones/child/ per year-at-risk [22] estimated as _mol_FOB by high resolution genotyping parasites. Despite the high endemicity, the incidence of clinical *P*. *vivax* malaria decreased from approximately 3.5 episodes to 1.5 episodes per year, suggesting acquisition of clinical immunity at young age [21]. In the present study, we identified for the first time a number of *P*. *vivax* coiled coil segments strongly associated with protection against clinical disease. Future studies will help to assess the value of the coiled coil antigens identified here as markers of acquired immunity or vaccine candidates.

## Materials and methods

### Study description

Blood samples were collected in 2006, in a rural area near Maprik, East Sepik Province of PNG [21], including children aged between 1–3 years at enrollment. Children were actively followed-up every 2 weeks, with clinical examination for malaria signs and symptoms, and blood-samples collected every 8 weeks for a period of 16 months. In addition, samples were collected through passive case detection at health centers. Malaria cases were confirmed by rapid diagnostic test (RDT) and all samples screened for *P*. *vivax*, *P*. *falciparum*, *P*. *malariae* and *P*. *ovale* by semi-quantitative post-PCR ligase detection reaction-fluorescent microsphere assay (LDR-FMA). All *P*. *vivax* positive samples were genotyped using the markers *msp1*F3 and MS16 [22] and all *P*. *falciparum* positive samples using the marker msp2 [24]. A total of 164 plasma samples collected at baseline, selected from children who completed follow-up were used to test antibody reactivity against 24 *P*. *vivax* coiled coil peptides. *P*. *vivax* clinical malaria episodes were defined as the presence of fever (i.e. axillary temperature >37.5°C) plus parasitemia (500 parasites/μL) [21].

### Ethics statement

Written informed consent was obtained from all parents or guardians prior to recruitment of each child. Scientific approval and ethical clearance for the study was obtained from the Medical Research and Advisory Committee (MRAC) of the Ministry of Health in PNG, the Institutional Review Board (IRB) from PNG Institute of Medical Research (IRB#1005) and the Human Research Ethics Committee of the Walter and Eliza Hall Institute.

### Antigens

Twenty-four coiled coil *P*. *vivax* polypeptides of 25 to 57 amino acids, previously described [20], were selected based on their reactivity with PNG and Colombia IgG antibodies (>30%), and were used to test the antibody reactivity of PNG samples. Briefly, peptides were synthesized by fluorenylmethoxycarbonyl (F-moc) solid-phase chemistry [25] using an Intavis AG Bioanalytical synthesizer (Germany) (Table 1). The resulting polypeptides were HPLC-purified. Purity (>80%) was confirmed by analytic C18 HPLC and mass spectrometry (MALDI-TOF, Applied Biosystems, Foster City, CA). All reagents were purchased from Fluka (Buchs, Switzerland) and Novabiochem (Laufelfingen, Switzerland).

**Table 1:** Coiled coil peptides protein of origin and sequences.

Peptide	Protein	MW	Sequence	Position
No	(aa nb)		(Plasmodb.org)	
*Pv*5	PVX_003585	2936	IADIKISLEKLKYEVKDKKDCLENV	203–227
*Pv*12	PVX_003585	5039	YKKELEEKAKIIEDLKDKICTLTNEVMDLKNVKNELAERDSSL	1023–1065
*Pv*27	PVX_113335	3169	KKQNAEKELSVLKKNYDAMSEEIEEIT	654–680
*Pv*40	PVX_119385	3634	NETIQRMSNSLLKYEQDIETYQNEVSTLTGK	675–705
*Pv*42	PVX_087730	3348	NTPDYYKKITTKLQNNINNVEEYINNITNDINILKSSID	154–192
*Pv*43	PVX_089660	4583	SVDINALNEQVKKLREELNKVTNEYDDFKNKLELLYQK	779–816
*Pv*45	PVX_123385	4333	KEVKVEVNEVGEEVNEVKEEVNEAKEEVIEKKEEMTE	650–686
*Pv*52	PVX_123480	3617	VEQVKKEINQINEQININETKITHLRNKIE	176–205
*Pv*63	PVX_118160	3658	NNEMDETLSKLKKDINKLNEKIQKYDNYVK	207–236
*Pv*81	PVX_118160	4442	NEMDETLSKLKKDINKLNEKIQKYDNYVKKKRKEID	208–243
*Pv*82.02	PVX_122740	6721	ETINQIDQKMEEIENNINLALEELKNLDQKILELQASFTCYENEIKQVIKKIEGLEK	862–918
*Pv*82.03	PVX_091910	6574	IEQLNTKMKNINENSNDSEHVNLAEFELKIAELKEDVNNINNMMKTFEMKFSALEK	471–526
*Pv*83	PVX_087730	4536	LQNNINNVEEYINNITNDINILKSSIDDERNERIIYNN	166–203
*Pv*90	PVX_00072	4164	TRRMHSELSDGNKELKKLKKNIVQSDVLNAQLELNI	63–98
*Pv*92	PVX_114000	4165	PDFDAYNEKLDSISESIDQVKKKIDNLQKEIKVANK	12–47
*Pv*95	PVX_11745	3512	EKGLKDLNDKIRNYDSIIENQKKELEHLK	145–173
*Pv*96.01	PVX_124060	6595	VEAVPENAEAAPENADPVHENAEAAPENAEPVHENAE	773–809
*Pv*96.03	PVX_084385	4482	DVQRIDTINKNISTINDDVDHINSNINNINDNLHKINSH	2051–2089
*Pv*101	PVX_085155	3554	NKLTEMRRKLKIIDEKVQSVYKAIHAVLNN	314–343
*Pv*106	PVX_114430	3441	KTIDQLDFEINDLNSKLKNYEKSVSQNKK	673–701
*Pv*112	PVX_092140	3502	KEMEKIDDQIDRIKNNIKKLNDDLNELTD	1143–1171
*Pv*121	PVX_094420	3506	LKFNSLKDILSKLLIEMKEHENQYNNLTE	151–179
*Pv*123	PVX_117855	3455	EKYSLIKEEIKYLNEDLDDLDNSVNVVKK	43–71
*Pv*145	PVX_100770	3864	MKHVNSLAFLYNEFKNNVEDLEKTYENFLKAL	1 32

### Coupling of polypeptides to fluorescent beads

Bio-Plex carboxylated beads (Bio-Rad) were covalently coated with the different coiled coil polypeptides following the manufacturer’s instructions (Bio-Plex Amine Coupling Kit). Briefly, microspheres (1.25 × 10^6^ beads/mL) were activated with a mixture of N-Hydroxysulfosuccinimide (5mg/mL) and N-(3-Dimethylaminopropyl)-N′-ethylcarbodiimide hydrochloride (5mg/mL) in H_2_O for 20 min at room temperature (RT) in the dark. Microspheres were then washed twice in 250 μL PBS, pH7.4, re-suspended in 500 μL of PBS, pH 7.4, and 1 μg of each polypeptide was added. Microspheres were incubated at 4°C overnight and re-suspended in 500 μL of blocking buffer (PBS + 1% BSA). After further incubation under rotation at RT for 30 minutes, beads were washed with PBS and stored at 4°C protected from light until use.

### Antibody assays

Coupled beads were used to analyze plasma reactivity as described previously [26]. Briefly, experimental plasma samples and controls were vortexed, centrifuged at maximum speed for 5–10 minutes in a microcentrifuge (Eppendorf), and diluted 1:100 in PBS plus tween 0.1% (PBT), 50 μL were then added to 50μL of previously mixed beads, and incubated at room temperature and protected from light for 30 min. After washing 3 times with 100 μL of PBT, aliquots of 100 μL of R-phycoerythrin conjugated anti-human IgG produced in Donkey (Jackson Immuno Research) diluted 1:100 were added and incubated for 15 min. Beads were washed and re-suspended in 125 μL of PBT and read on a Bio-Plex 200. Each plate also contained a blank (wells containing only beads and PBT). Negative control consisted on a pool of plasma samples from individuals never exposed to malaria diluted 1:100, and positive control consisted on serial dilutions (1:50–1:25,600) of pooled serum (n = 20) from PNG immune adults.

### Statistical analysis

Luminex median fluorescence intensity (MFI) values were converted into arbitrary antibody units based on the parameters estimated from a standard curve made with dilutions of the highly-immune PNG positive control pool, as previously described [27]. Antibody units ranged from 1.95x10-5 (i.e., equivalent to 1:51,200 dilution of the immune pool) to 0.02 (1:50). A cut-off for positivity was calculated as three standard deviations above the dilution value of negative controls for each antigen. Individuals were considered sero-positive if the dilutions of the experimental plasma samples were higher than the cut-off of negative controls cut-off. All samples were tested in duplicates in two independent experiments. Differences in the prevalence of IgG antibodies with age, infection status and exposure were assessed using Fisher’s exact test. The Spearman rank correlation coefficient was used to investigate the relationship among antibody responses to the coiled coil antigens. A heat map was created using the corrplot function in R. The association between antibody levels to *P*. *vivax* coiled coil antigens and protection against clinical malaria (defined as fever and parasite density >500/μL) [21], as well as the association between number of recognized antigens and the risk of *P*. *vivax* clinical episodes was assessed using negative binomial GEE models with exchangeable correlation structure and semi-robust variance estimator [27, 28]. IgG levels were classified into terciles and analyses done by comparing children with low versus medium and high antibody levels. All GEE models were adjusted for seasonal trends, village of residency, age and individual differences in exposure (_mol_FOB). Multivariate analysis with all antigens was also performed using a backward stepwise regression for the selection of the final model. Analyses were performed using STATA version 12 (StataCorp) or R version 3.2.1 [29].

## Results

### Prevalence of IgG antibodies and their association with age, infection status and village of residency

**Fig 1:**
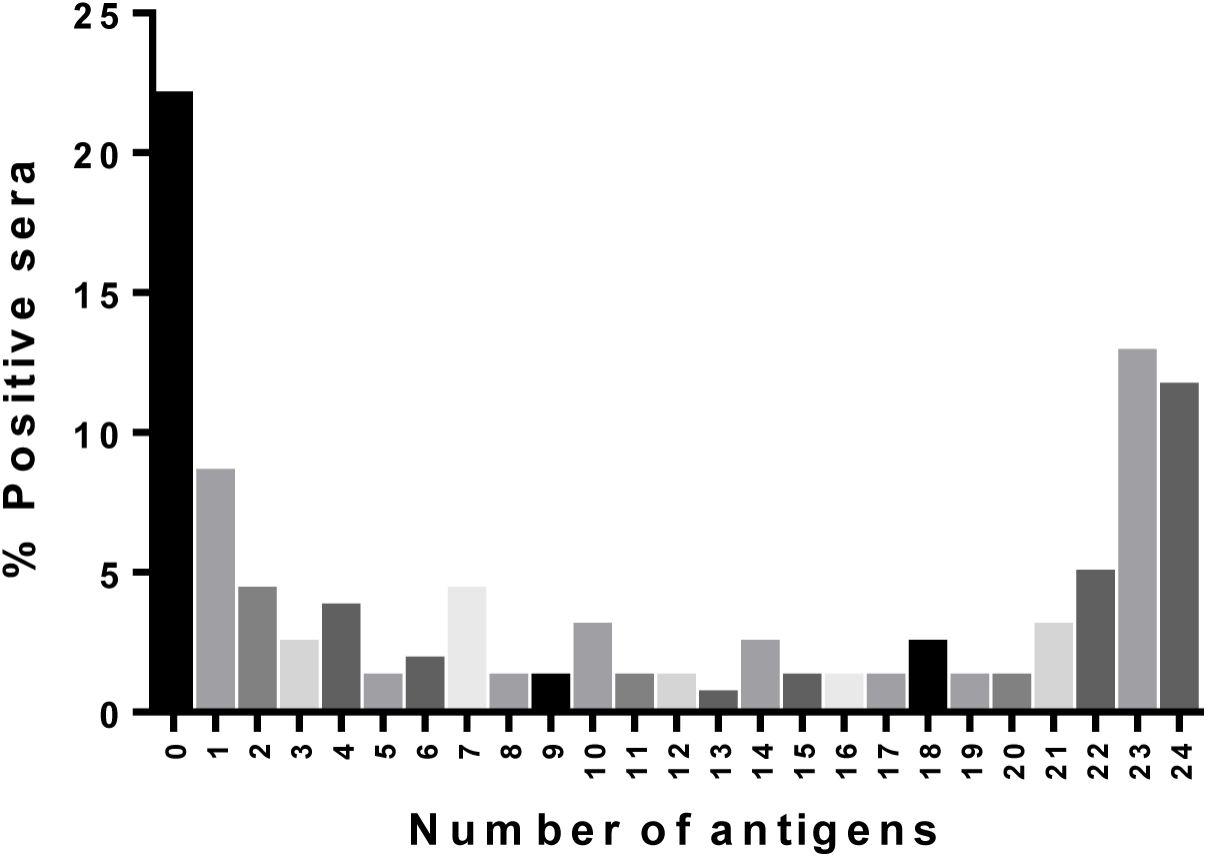
Positivity to different α-helical coiled coil peptides. Data are plotted as the percentage of individuals who are antibody positive for 0–24 of the antigens tested.

As expected based on the selection criteria for these 24 peptides, plasma samples collected at enrollment were reactive to all 24 peptides, with variable frequencies (Table 2). Eight peptides had the highest percentage of responders with >50% of children reactive (*Pv*121; *Pv*82.02 *Pv*96.01; *Pv*82.03; *Pv*95; *Pv*40; *Pv*52 and *Pv*12), whereas only one peptide (*Pv*90) had < 20% responders. Overall, 21/164 (13%) samples had antibodies reactive to at least 23 peptides, whereas 36/164 (22%) samples showed no reaction to any of the peptides tested (Fig 1). All plasma samples (n = 19) that reacted with all antigens were infected at sampling time, although there were a few samples (8/36) infected at sampling that did not react with any of the antigens.

**Table 2:** Dilution of total IgG response to the different coiled coil peptides.

Antigen	Median dilution (IQR)	Cut off^a^	Percentage of responders^b^ (n)
Pv5	3.5E-03 (1.9E-03, 7.1E-03)	4.4E-03	39.6% (65)
**Pv12**	**3.4E-03 (2.1E-03, 6.6E-03)**	**3.4E-03**	**50.0% (82)**
Pv27	1.0E-03 (6.3E-04, 1.6E-03)	1.3E-03	34.8% (57)
**Pv40**	**4.4E-03 (2.6E-03, 9.6E-03)**	**4.3E-03**	**51.2% (84)**
Pv42	4.0E-03 (2.5E-03, 7.1E-03)	4.3E-03	46.3% (76)
Pv43	2.2E-03 (1.3E-03, 4.0E-03)	2.5E-03	43.9% (72)
Pv45	3.7E-03 (2.6E-03, 6.8E-03)	4.5E-03	37.2% (61)
**Pv52**	**2.0E-03 (1.3E-03, 3.7E-03)**	**2.0E-03**	**50.6% (83)**
Pv63	6.9E-04 (4.6E-04, 1.4E-03)	8.4E-04	43.3% (71)
Pv81	2.6E-03 (1.8E-03, 4.9E-03)	3.1E-03	40.2% (66)
**Pv82.02**	**3.6E-03 (2.3E-03, 7.4E-03)**	**2.8E-03**	**64.0% (105)**
**Pv82.03**	**2.5E-03 (1.4E-03, 4.0E-03)**	**2.3E-03**	**53.0% (87)**
Pv83	3.2E-03 (2.1E-03, 6.6E-03)	3.6E-03	43.9% (72)
Pv90	3.7E-03 (2.3E-03, 6.6E-03)	8.5E-03	18.9% (31)
Pv92	2.5E-03 (1.6E-03, 4.4E-03)	3.2E-03	35.4% (58)
**Pv95**	**1.0E-03 (6.1E-04, 1.8E-03)**	**9.7E-04**	**52.4% (86)**
**Pv96.01**	**3.9E-03 (2.2E-03, 8.2E-03)**	**3.3E-03**	**56.1% (92)**
Pv96.03	7.8E-04 (5.0E-04, 1.7E-03)	8.1E-04	47.0% (77)
Pv101	3.5E-03 (2.0E-03, 6.5E-03)	4.3E-03	40.9% (67)
Pv106	3.8E-03 (2.2E-03, 7.7E-03)	4.1E-03	47.6% (78)
Pv112	5.5E-03 (3.7E-03, 1.4E-02)	5.9E-03	47.6% (78)
**Pv121**	**4.2E-03 (2.7E-03, 8.1E-03)**	**3.1E-03**	**64.6% (106)**
Pv123	5.0E-03 (3.2E-03, 1.0E-02)	5.5E-03	43.3% (71)
Pv145	4.5E-03 (2.6E-03, 1.0E-02)	4.5E-03	49.4% (81)

^a^The cut-off for positivity was determined as the mean+3 standard deviations of negative control plasma samples (Australian residents) included in each assay.

^b^Responders defined as individuals whose plasma dilution was above the cut-off for positivity for a given antigen.

Bold peptides correspond to those reacting with >50% of the tested sera

There were some differences between Ilahita and Sunuhu villages regarding malaria prevalence and age at enrollment [21]. The percentage of infected children at enrollment was higher in Sunuhu than in Ilahita (33/62; 53% and 39/102; 38% respectively, P = 0.033). For this reason, reactivity of total IgG from children in these two villages were compared. Children from Sunuhu were more likely to have IgG antibodies reactive to 22 peptides, although significant differences were observed only for seven of them (Pv12, Pv63, Pv82.03, Pv95, Pv106, Pv121 and Pv145) (S1 Table). Higher positivity was observed in two hamlets (Ilahita 5 and Sunuhu 1) for 23 of the 24 antigens (S2 Table).

A positive correlation was observed between antibody positivity to 23 α-helical coiled coil peptides and *P*. *vivax* infection at enrollment (Table 3), with stronger association for peptides *Pv*5, *Pv*83, *Pv*121, *Pv*101, *Pv*52, *Pv*81, *Pv*106, *Pv*145, *Pv*82.02 and *Pv*96.03 (P = 0.001–0.010). Only one peptide (*Pv*90) had no association with infection status (Table 3). There was no significant association between antibody levels to any of the α-helical coiled coil peptides and age or exposure measured by genotyping during follow-up (S3 and S4 Tables).

**Table 3:** Reactivity of antibodies at enrollment based on status of infection.

Antigen	Infected^a^ (%)		Non-infected (%)		OR	P-value^d^
	Positive^b^	Negative^c^	Positive^b^	Negative^c^		
Pv5	39 (24)	33 (20)	26 (16)	66 (40)	3.00	0.001
Pv12	43 (26)	29 (18)	39 (24)	53 (32)	2.02	0.028
Pv27	32 (20)	40 (24)	25 (15)	67 (41)	2.14	0.021
Pv40	44 (27)	28 (17)	40 (24)	52 (32)	2.04	0.025
Pv42	41 (25)	31 (19)	35 (21)	57 (35)	2.15	0.016
Pv43	39 (24)	33 (20)	33 (20)	59 (36)	2.11	0.019
Pv45	32 (20)	40 (24)	29 (18)	63 (38)	1.74	0.089
Pv52	46 (28)	26 (16)	37 (23)	55 (34)	2.63	0.003
Pv63	38 (23)	34 (21)	33 (20)	59 (36)	2.00	0.030
Pv81	38 (23)	34 (21)	28 (17)	64 (39)	2.55	0.004
Pv82.02	54 (33)	18 (11)	51 (31)	41 (25)	2.41	0.010
Pv82.03	46 (28)	26 (16)	41 (25)	51 (31)	2.20	0.014
Pv83	42 (26)	30 (18)	30 (18)	62 (38)	2.89	0.001
Pv90	15 (9)	57 (35)	16 (10)	76 (46)	1.25	0.576
Pv92	33 (20)	39 (24)	25 (15)	67 (41)	2.27	0.013
Pv95	44 (27)	28 (17)	42 (26)	50 (30)	1.87	0.049
Pv96.01	47 (29)	25 (15)	45 (27)	47 (29)	1.96	0.036
Pv96.03	42 (26)	30 (18)	35 (21)	57 (35)	2.28	0.010
Pv101	39 (24)	33 (20)	28 (17)	64 (39)	2.70	0.002
Pv106	43 (26)	29 (18)	35 (21)	57 (35)	2.41	0.006
Pv112	41 (25)	31 (19)	37 (23)	55 (34)	1.97	0.033
Pv121	56 (34)	16 (10)	50 (30)	42 (26)	2.94	0.002
Pv123	38 (23)	34 (21)	33 (20)	59 (36)	2.00	0.030
Pv145	44 (27)	28 (17)	37 (23)	55 (34)	2.34	0.008

^a^*P*. *vivax* infection status was determined by post-PCR LDR-FMA at enrollment.

^b^Samples positive for infection at enrollment (n = 72)

^c^Samples negative for *P*. *vivax* infection at enrollment (n = 92)

^d^P values ≤0.05 were considered significant. P-value calculated by Fisher’s exact test

**Fig 2:**
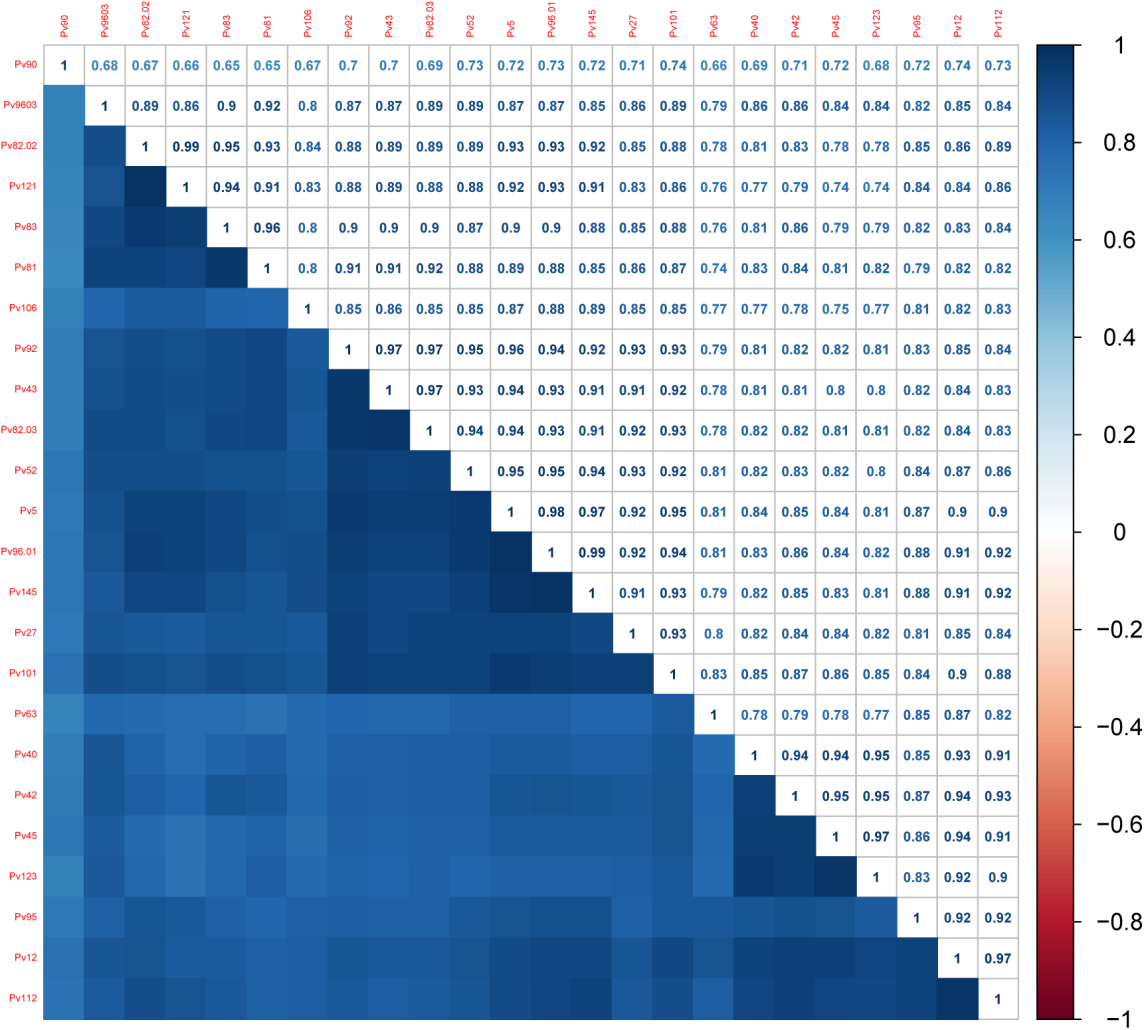
Heat map representation of correlation of antibody response between coiled coil antigens. The heat map colors correspond to correlations grading from -1 (negative correlation, red), no correlation (white) to 1 (positive correlation, blue).

Arbitrary antibody units observed to all coiled coil antigens were positively correlated with each other (Fig 2). The most correlated were Pv82.02-Pv121 (r = 0.99), Pv145-Pv96.01 (r = 0.99), Pv5-Pv96.01 (r = 0.98), and the less correlated with the other antigens was Pv96.

### Association between antibody titer and prospective risk of *P*. *vivax* malaria

**Fig 3:**
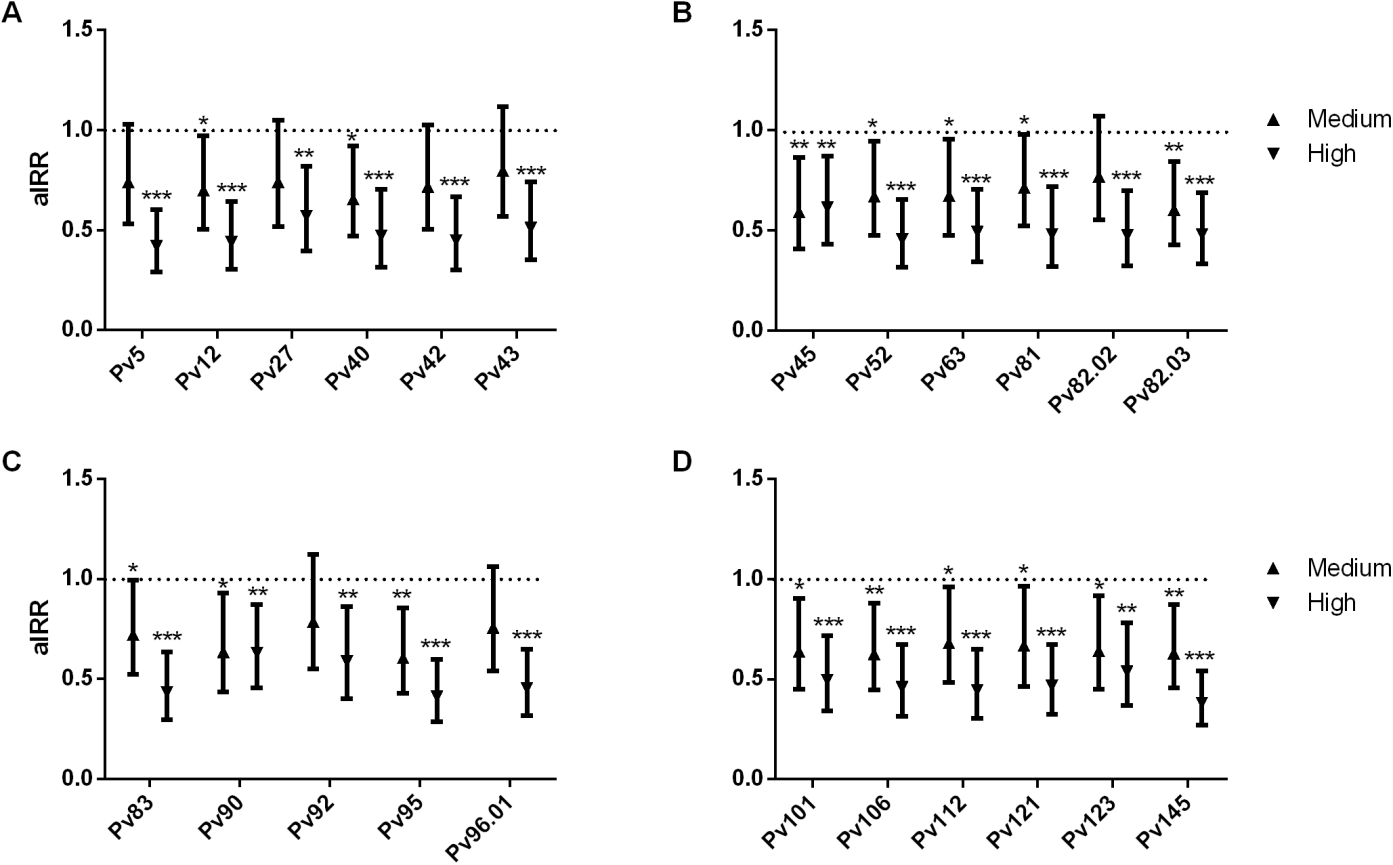
IgG to *P*. *vivax* coiled coil antigens and risk of clinical malaria in PNG children. Data are plotted as incidence rate ratios and 95% confidence intervals, adjusted for exposure (molFOB), age, season, and village of residency. P values <0.05 were considered significant. *P > 0.05 to 0.01; ** P > 0.01 to 0.001; ***P < 0.001.

During the 16 months of follow-up, children experienced an incidence rate of 1.28 (95% CI 1.08–1.52) clinical malaria episodes. An adjusted GEE model was applied to test whether responses to the coiled coil antigens were associated with risk of clinical disease. Significantly lower risk of clinical *P*. *vivax* malaria was associated with high levels of IgG for all coiled coil antigens (Table 4) (adjusted incidence rate ratio (aIRR) ranging from 0.351 to 0.598). When adjusting for confounders such as age, village of residence, seasonality and individual differences in exposure, IgG levels to each of coiled coil antigens remained associated with protection with aIRR ranging from 0.314 to 0.548. Medium and high levels of IgG to 16 coiled coil fragments were associated with protection. For Pv5, Pv27, Pv42, Pv43, Pv82.02, Pv92 and Pv96.01, only high (but nor medium) IgG levels were significantly associated with protection (Fig 3; S5 Table). In general, there was a strong association between increasing antibody levels and protection for all coiled coil fragments except Pv45 and Pv90. In addition, the association between the number of recognized antigens and the risk of *P*. *vivax* clinical episodes was assessed, identifying that for each additional recognized antigen there was a reduction in *P*. *vivax* clinical risk (aIRR: 0.970, P<0.001, 95% CI: 0.953–0.987). Finally, when multivariate analyses and backward stepwise regression were performed, only antibody levels to Pv95 (aIRR(multi) = 0.35, p<0.001, 95% CI: 0.209, 0.604) remained significantly associated with a reduced risk of *P*. *vivax* clinical disease.

**Table 4:** Association between IgG levels to *P*. *vivax* antigens and protection against clinical malaria (parasite density >500/μl of blood).

Antigen	^a^IRR	^b^95% CI	^c^P-value	^d^aIRR	^b^95% CI	^c^P-value
Pv5	0.398	(0.250, 0.633)	<0.001	0.385	(0.249, 0.596)	<0.001
Pv12	0.427	(0.248, 0.734)	0.0021	0.385	(0.237, 0.625)	<0.001
Pv27	0.395	(0.208, 0.749)	0.0044	0.414	(0.231, 0.740)	<0.001
Pv40	0.425	(0.275, 0.657)	<0.001	0.402	(0.262, 0.618)	<0.001
Pv42	0.399	(0.231, 0.689)	<0.001	0.377	(0.223, 0.639)	<0.001
Pv43	0.48	(0.303, 0.761)	0.0018	0.441	(0.286, 0.680)	<0.001
Pv45	0.451	(0.256, 0.794)	0.0058	0.416	(0.252, 0.686)	<0.001
Pv52	0.404	(0.240, 0.681)	<0.001	0.397	(0.250, 0.631)	<0.001
Pv63	0.538	(0.288, 1.007)	0.0525	0.479	(0.319, 0.719)	<0.001
Pv81	0.441	(0.272, 0.715)	<0.001	0.405	(0.252, 0.650)	<0.001
Pv82.02	0.374	(0.234, 0.597)	<0.001	0.366	(0.230, 0.581)	<0.001
Pv82.03	0.419	(0.255, 0.690)	<0.001	0.413	(0.261, 0.653)	<0.001
Pv83	0.396	(0.249, 0.630)	<0.001	0.39	(0.246, 0.619)	<0.001
Pv90	0.598	(0.357, 1.000)	0.05	0.548	(0.356, 0.844)	0.0063
Pv92	0.429	(0.270, 0.680)	<0.001	0.411	(0.268, 0.630)	<0.001
Pv95	0.375	(0.219, 0.644)	<0.001	0.338	(0.206, 0.556)	<0.001
Pv96.01	0.425	(0.270, 0.669)	<0.001	0.401	(0.258, 0.623)	<0.001
Pv96.03	0.475	(0.304, 0.743)	0.0011	0.468	(0.305, 0.719)	<0.001
Pv101	0.454	(0.276, 0.749)	0.0020	0.415	(0.259, 0.665)	<0.001
Pv106	0.526	(0.307, 0.901)	0.0194	0.479	(0.299, 0.766)	0.0021
Pv112	0.351	(0.209, 0.587)	<0.001	0.314	(0.190, 0.521)	<0.001
Pv121	0.384	(0.241, 0.610)	<0.001	0.372	(0.236, 0.586)	<0.001
Pv123	0.475	(0.293, 0.770)	0.0025	0.43	(0.271, 0.680)	<0.001
Pv145	0.416	(0.266, 0.649)	<0.001	0.377	(0.242, 0.588)	<0.001

^a^IRRs incidence rate ratio, are derived from crude negative binomial GEE models. GEE, generalized estimating equation.

^b^95% CI, 95% confidence interval.

^c^P-values ≤ 0.05 were considered statistically significant.

^d^aIRRs adjusted incidence rate ratios, are negative binomial GEE model estimates adjusted for age, village of residence, seasonality, and individual differences in exposure (_mol_FOB).

IgG responses to any *P*. *vivax* coiled coil antigens were not associated with protection against *P*. *falciparum* clinical episodes, suggesting that these associations observed are species-specific (S6 Table).

## Discussion

Repeated exposure to malaria infection results in development of clinical immunity, which is a multifactorial process where antibodies play a major role [30]. Both polyclonal and monoclonal antibodies have shown the potential to prevent malaria infections or to completely clear parasitemia [31, 32]. *Plasmodium* coiled coil domains have been previously been proposed as potential targets for human malaria vaccine development, since such domains fold into stable structures that are capable of eliciting antibodies reactive against parasite functional epitopes, and are in general non- or little polymorphic [17, 33].

Down selection of *P*. *vivax* antigens could represent a challenge due to the technical difficulties and limitations presented by the lack of an *in vitro* culture system for this parasite species. Previously, naturally acquired antibodies to the *P*. *vivax* merozoite surface protein 1 (Pv-MSP1) [34] and binding-inhibitory antibodies response to *P*. *vivax* Duffy binding protein (PvDBP) [35] have been associated with reduced risk of *P*. *vivax* clinical manifestations in individuals from the Brazilian Amazon region. In addition, the contribution of antibody titers to the acquisition of protection against clinical disease has been assessed in a cohort of children from PNG. In this population, immunity against clinical *P*. *vivax* malaria was acquired at a young age with a 3-fold decrease in episodes between the ages of 1 and 4 years [21, 36, 37]. In previous studies using this cohort, it have been demonstrated a strong association between specific IgG to merozoite surface proteins PvMSP3a, PvMSP9, [28], three novel merozoite proteins (PVX_081550, P12 and P41, [27]) as well as the reticulocyte specific binder RBP2b and RBP1a and clinical protection [38].

Here, by using the same PNG cohort we demonstrated that *P*. *vivax* coiled coil antigens are also targeted by natural acquired immunity and reactive with plasma from all *Plasmodium* species including both *P*. *vivax* and *P*. *falciparum* exposed and infected children. This antibody reactivity observed at recruitment time was associated with acquisition of protective immunity against only *P*. *vivax* clinical disease. It was found that children with high antibody levels to all the coiled coil antigens had 2–3 fold lower risk of clinical *P*. *vivax* malaria during follow-up. In addition, a positive association between the number of recognized antigens and protection against *P*. *vivax* clinical episodes was also found. This corroborates the role of acquired immunity as explanation for the decrease in incidence of *P*. *vivax* clinical disease with an increase in age, despite constant exposure [21, 36, 37]. Multivariate analysis revealed antibody levels to Pv95 as the only antigen significantly associated with a reduced risk of clinical malaria, which may indicate that this fragment is particularly targeted by natural immunity or a good marker of immunity. Given the high correlation between antibody responses to the coiled coil fragments, it is difficult to differentiate co-acquisition of antibodies from cross reactivity in multivariate analyses. This correlation is observed even though most of the antigens are derived from completely different proteins with limited sequence homology among the peptides, except for Pv63 and Pv81 that show high level of homology (98%) [20]. Although cross-reactivity is likely, 24 peptides were tested using 164 individuals samples and for several individual donors a unique set of peptides was recognized (with the only exceptions for those that recognized none or most of the peptides) (S1 Fig). In addition, by using affinity-purified antibodies no cross-reactivity was observed between the *P*. *falciparum* protein sequences orthologous to *P*. *vivax* [17]. Therefore, further research in different populations exposed to *P*. *vivax* is however warranted to confirm this association.

Serum samples from PNG were reactive to all *P*. *vivax* coiled coil peptides tested, and in most cases, prevalence was comparable to that previously observed using adult’s sera from a different PNG region [20]. Moreover, about 25% of children were infected at baseline, and their plasma showed reactivity to most of the antigens. These data suggest that some of these antigens could be markers of recent exposure.

Increased reactivity to *P*. *vivax* coiled coil peptides was observed in children living in villages where exposure to *P*. *falciparum* is higher (Ilahita 5 and Sunuhu 1) [21]. This observation could be explained by the high homology observed between the *P*. *falciparum* and *P*. *vivax* coiled coil antigens [20]. Indeed, cross-reactivity to *P*. *vivax* peptides was observed in sera from African donors (Supplementary results, S7 Table). However, in the PNG cohort there was no association between antibody response to *P*. *vivax* antigens and protection from clinical *P*. *falciparum* malaria. Even though the numbers of clinical episodes with *P*. *falciparum* and *P*. *vivax* were similar, there was no evidence of acquisition of clinical immunity to *P*. *falciparum* in the age group covered by this cohort [21]. These results correlate with previous studies using this study cohort, where no significant associations was observed between antibody responses to any of the P. vivax proteins and risk of *P*. *falciparum* clinical episodes [27, 28]. This could be explained by differences in the age of immunity acquisition, the decrease in risk of infection to *P*. *falciparum* is only seen in adolescence and early adulthood [39].

Data presented here, along with those previously published [20], point to the 24 selected coiled coil *P*. *vivax* antigens as good markers of acquired immunity to *P*. *vivax* and to an important potential source of malaria vaccine candidates. Further insight may be provided by studies in animal models i.e. primates to determine experimentally the protective efficacy of these antibodies against *P*. *vivax* blood infection as well as to further assess the reactivity between peptides and select the most promising candidates for vaccine development.

## Supporting information

S1 Fig Reactivity pattern of sera samples against the *P*. *vivax* coiled coil fragments. (PDF)

S1 Table Prevalence of responders to *P*. *vivax* coiled coil fragments in young children from Ilahita and Sunuhu villages. (DOCX)

S2 Table Antibody reactivity at enrollment by age group. (DOCX)

S3 Table Prevalence of responders reactive to *P*. *vivax* coiled coil fragments in volunteers from the different tested villages in PNG. (XLSX)

S4 Table Reactivity of antibodies at enrollment and molecular Force of Infection. (XLSX)

S5 Table Association between IgG levels to *P*. *vivax* antigens and protection against clinical malaria (XLSX)

S6 Table Association between IgG levels to *P*. *vivax* antigens and protection against *P*. *falciparum* clinical disease (with parasite density >2500/μl of blood). (DOCX)

S7 Table Prevalence of antibody responders to *P*. *vivax* coiled coil fragments in donors from Burkina Faso. (DOCX)
