# Supplementary material for: Natural immune response to Plasmodium vivax alpha-helical coiled coil protein motifs and its association with the risk of P. vivax malaria
Source: PLoS One. 2017 Jun 26;12(6):e0179863. doi: 10.1371/journal.pone.0179863 (PMC5484505; doi:10.1371/journal.pone.0179863)
Supplement: S1 Fig — (PDF) [file pone.0179863.s001.pdf]

Sample code	#	Pv121	Pv82.02	Pv96.01	Pv82.03	Pv95	Pv40	Pv52	Pv12	Pv145	Pv106	Pv112	Pv96.03	Pv42	Pv43	Pv83	Pv63	Pv123	Pv101	Pv81	Pv5	Pv45	Pv92	Pv27	Pv90
XOA2	24	0,015	0,015	0,020	0,020	0,005	0,020	0,020	0,020	0,020	0,020	0,020	0,009	0,020	0,020	0,015	0,004	0,020	0,020	0,015	0,020	0,020	0,020	0,009	0,020
XOA6	24	0,015	0,015	0,020	0,020	0,018	0,020	0,020	0,020	0,020	0,020	0,020	0,015	0,020	0,020	0,015	0,018	0,020	0,020	0,015	0,020	0,020	0,020	0,020	0,020
XOA12	24	0,016	0,014	0,014	0,008	0,002	0,015	0,005	0,009	0,016	0,018	0,019	0,002	0,010	0,006	0,014	0,002	0,015	0,009	0,009	0,013	0,008	0,007	0,003	0,009
XOA26	24	0,008	0,007	0,011	0,010	0,001	0,007	0,004	0,005	0,011	0,006	0,011	0,002	0,006	0,005	0,007	0,001	0,008	0,007	0,006	0,009	0,006	0,006	0,002	0,011
XOA63	24	0,009	0,008	0,008	0,005	0,002	0,012	0,006	0,012	0,009	0,010	0,014	0,001	0,012	0,005	0,006	0,002	0,012	0,005	0,005	0,007	0,011	0,005	0,001	0,020
XOA74	24	0,017	0,017	0,016	0,013	0,008	0,020	0,009	0,017	0,018	0,015	0,020	0,006	0,016	0,014	0,017	0,003	0,017	0,016	0,015	0,015	0,017	0,013	0,008	0,010
XOA83	24	0,008	0,007	0,010	0,003	0,002	0,010	0,003	0,008	0,014	0,007	0,015	0,002	0,008	0,003	0,006	0,001	0,008	0,007	0,004	0,007	0,007	0,003	0,002	0,020
XOA95	24	0,009	0,009	0,009	0,004	0,002	0,013	0,004	0,013	0,010	0,017	0,015	0,002	0,013	0,004	0,009	0,001	0,012	0,007	0,006	0,008	0,013	0,005	0,002	0,019
XOA105	24	0,014	0,013	0,020	0,015	0,003	0,020	0,010	0,015	0,020	0,015	0,020	0,014	0,020	0,020	0,015	0,005	0,020	0,020	0,013	0,018	0,018	0,014	0,004	0,012
XOA118	24	0,017	0,016	0,015	0,011	0,002	0,019	0,007	0,016	0,016	0,010	0,019	0,004	0,016	0,009	0,017	0,001	0,020	0,013	0,015	0,014	0,017	0,011	0,004	0,014
XOA122	24	0,014	0,014	0,020	0,020	0,004	0,020	0,020	0,017	0,020	0,020	0,020	0,007	0,020	0,020	0,014	0,003	0,020	0,020	0,014	0,020	0,020	0,020	0,009	0,015
XOA130	24	0,020	0,020	0,019	0,014	0,004	0,020	0,014	0,020	0,020	0,016	0,020	0,009	0,020	0,015	0,020	0,003	0,020	0,020	0,020	0,017	0,020	0,014	0,008	0,020
XOA151	24	0,020	0,020	0,020	0,018	0,006	0,020	0,015	0,020	0,020	0,020	0,020	0,012	0,020	0,016	0,020	0,004	0,020	0,020	0,020	0,018	0,020	0,015	0,012	0,020
XOA157	24	0,015	0,015	0,020	0,020	0,020	0,020	0,020	0,020	0,020	0,020	0,020	0,015	0,020	0,020	0,015	0,020	0,020	0,020	0,015	0,020	0,020	0,020	0,020	0,020
XOA158	24	0,015	0,015	0,014	0,010	0,006	0,020	0,008	0,020	0,012	0,013	0,020	0,013	0,020	0,008	0,015	0,004	0,020	0,016	0,015	0,015	0,020	0,009	0,004	0,020
XOA167	24	0,010	0,009	0,015	0,011	0,003	0,017	0,011	0,009	0,017	0,015	0,020	0,002	0,010	0,009	0,009	0,004	0,017	0,014	0,007	0,013	0,010	0,009	0,004	0,020
XOA171	24	0,015	0,015	0,020	0,020	0,011	0,020	0,020	0,020	0,020	0,020	0,020	0,014	0,020	0,020	0,015	0,010	0,020	0,020	0,015	0,020	0,020	0,020	0,020	0,020
XOA174	24	0,010	0,010	0,016	0,010	0,002	0,018	0,006	0,011	0,012	0,009	0,019	0,003	0,016	0,008	0,010	0,002	0,020	0,014	0,009	0,012	0,015	0,010	0,003	0,010
XOA179	24	0,011	0,011	0,013	0,008	0,004	0,020	0,006	0,020	0,012	0,007	0,020	0,005	0,020	0,007	0,011	0,003	0,020	0,012	0,011	0,014	0,020	0,008	0,003	0,015
XOA10	23	0,005	0,004	0,006	0,004	0,001	0,006	0,003	0,004	0,006	0,006	0,006	0,001	0,005	0,004	0,005	0,001	0,006	0,009	0,005	0,005	0,005	0,006	0,002	0,004
XOA15	23	0,012	0,008	0,007	0,004	0,001	0,012	0,004	0,004	0,009	0,010	0,014	0,001	0,007	0,004	0,006	0,001	0,012	0,005	0,006	0,007	0,005	0,004	0,001	0,005
XOA22	23	0,012	0,014	0,020	0,020	0,001	0,015	0,015	0,016	0,018	0,010	0,015	0,004	0,013	0,019	0,013	0,001	0,016	0,013	0,015	0,020	0,013	0,020	0,005	0,005
XOA28	23	0,014	0,013	0,010	0,005	0,002	0,012	0,004	0,006	0,011	0,007	0,014	0,003	0,012	0,006	0,012	0,001	0,012	0,008	0,012	0,007	0,011	0,005	0,002	0,007
XOA29	23	0,010	0,009	0,011	0,008	0,001	0,012	0,005	0,007	0,011	0,007	0,013	0,002	0,008	0,008	0,010	0,001	0,014	0,005	0,008	0,011	0,009	0,011	0,002	0,004
XOA30	23	0,010	0,011	0,014	0,015	0,002	0,007	0,008	0,006	0,014	0,015	0,011	0,002	0,006	0,009	0,010	0,001	0,007	0,010	0,008	0,013	0,005	0,010	0,004	0,005
XOA39	23	0,008	0,007	0,015	0,008	0,005	0,013	0,007	0,012	0,019	0,015	0,015	0,002	0,013	0,008	0,007	0,001	0,013	0,013	0,006	0,016	0,013	0,007	0,004	0,007
XOA41	23	0,020	0,018	0,017	0,007	0,003	0,015	0,007	0,016	0,019	0,018	0,020	0,003	0,015	0,007	0,014	0,001	0,014	0,012	0,010	0,015	0,013	0,009	0,004	0,005
XOA43	23	0,008	0,006	0,007	0,004	0,003	0,008	0,005	0,020	0,010	0,009	0,014	0,002	0,006	0,004	0,007	0,001	0,008	0,005	0,004	0,006	0,005	0,004	0,002	0,004
XOA49	23	0,007	0,006	0,008	0,003	0,002	0,009	0,003	0,009	0,011	0,007	0,013	0,001	0,007	0,003	0,005	0,003	0,009	0,007	0,004	0,006	0,007	0,004	0,003	0,005
XOA59	23	0,017	0,016	0,018	0,014	0,002	0,016	0,011	0,010	0,019	0,014	0,020	0,004	0,011	0,014	0,011	0,002	0,020	0,011	0,013	0,018	0,011	0,017	0,004	0,004
XOA58	23	0,014	0,012	0,017	0,015	0,003	0,020	0,010	0,009	0,016	0,015	0,020	0,003	0,010	0,012	0,010	0,001	0,018	0,010	0,010	0,016	0,009	0,016	0,006	0,005
XOA68	23	0,005	0,004	0,013	0,005	0,003	0,012	0,005	0,017	0,014	0,011	0,016	0,001	0,010	0,005	0,004	0,013	0,012	0,010	0,003	0,009	0,009	0,008	0,002	0,011
XOA75	23	0,012	0,010	0,011	0,003	0,005	0,008	0,004	0,009	0,013	0,005	0,020	0,001	0,007	0,003	0,005	0,001	0,007	0,005	0,004	0,007	0,007	0,005	0,001	0,020
XOA77	23	0,017	0,016	0,017	0,010	0,003	0,018	0,008	0,017	0,020	0,017	0,020	0,003	0,016	0,009	0,016	0,003	0,016	0,012	0,012	0,016	0,015	0,012	0,003	0,006
XOA101	23	0,008	0,008	0,008	0,006	0,001	0,009	0,004	0,005	0,009	0,006	0,009	0,003	0,008	0,006	0,009	0,001	0,017	0,009	0,008	0,009	0,008	0,007	0,002	0,004
XOA113	23	0,007	0,007	0,008	0,006	0,001	0,015	0,004	0,006	0,009	0,005	0,008	0,002	0,007	0,006	0,007	0,001	0,015	0,010	0,008	0,007	0,007	0,006	0,002	0,005
XOA131	23	0,013	0,010	0,009	0,005	0,003	0,012	0,004	0,007	0,011	0,009	0,014	0,002	0,007	0,005	0,007	0,002	0,012	0,005	0,005	0,008	0,005	0,006	0,003	0,005
XOA144	23	0,011	0,009	0,011	0,011	0,002	0,014	0,006	0,007	0,012	0,007	0,013	0,002	0,010	0,008	0,011	0,001	0,014	0,013	0,009	0,011	0,011	0,010	0,003	0,008
XOA160	23	0,020	0,020	0,013	0,011	0,005	0,014	0,006	0,008	0,013	0,007	0,014	0,003	0,009	0,011	0,014	0,001	0,014	0,006	0,018	0,013	0,009	0,014	0,002	0,004
XOA188	23	0,007	0,006	0,006	0,004	0,002	0,010	0,003	0,006	0,009	0,005	0,011	0,001	0,007	0,005	0,005	0,001	0,012	0,006	0,004	0,006	0,008	0,004	0,001	0,007
XOA4	22	0,007	0,007	0,005	0,004	0,001	0,008	0,003	0,006	0,006	0,003	0,009	0,002	0,007	0,003	0,007	0,001	0,011	0,006	0,006	0,005	0,008	0,004	0,001	0,005
XOA38	22	0,007	0,006	0,009	0,003	0,002	0,009	0,003	0,007	0,010	0,013	0,013	0,001	0,015	0,003	0,008	0,001	0,008	0,007	0,006	0,006	0,006	0,003	0,001	0,005
XOA47	22	0,011	0,009	0,009	0,003	0,002	0,006	0,003	0,008	0,011	0,010	0,020	0,001	0,007	0,006	0,005	0,001	0,007	0,005	0,004	0,007	0,004	0,004	0,001	0,005
XOA48	22	0,020	0,020	0,019	0,008	0,009	0,006	0,006	0,008	0,018	0,011	0,016	0,003	0,006	0,010	0,011	0,002	0,005	0,011	0,007	0,015	0,004	0,008	0,002	0,017
XOA62	22	0,014	0,011	0,011	0,005	0,002	0,007	0,004	0,006	0,013	0,010	0,017	0,002	0,009	0,004	0,010	0,001	0,006	0,007	0,007	0,010	0,005	0,005	0,002	0,005
XOA66	22	0,004	0,004	0,008	0,003	0,002	0,009	0,004	0,012	0,014	0,015	0,017	0,002	0,009	0,003	0,003	0,004	0,010	0,007	0,002	0,008	0,008	0,004	0,002	0,012
XOA80	22	0,020	0,016	0,015	0,006	0,002	0,005	0,009	0,007	0,016	0,013	0,014	0,001	0,006	0,005	0,010	0,001	0,004	0,009	0,006	0,011	0,003	0,006	0,002	0,020
XOA109	22	0,004	0,004	0,006	0,003	0,001	0,008	0,002	0,005	0,006	0,004	0,007	0,001	0,006	0,003	0,005	0,001	0,010	0,007	0,004	0,005	0,007	0,004	0,002	0,005
XOA35	21	0,007	0,006	0,010	0,002	0,002	0,011	0,003	0,006	0,012	0,006	0,012	0,001	0,010	0,002	0,006	0,001	0,010	0,006	0,004	0,006	0,007	0,003	0,001	0,004
XOA44	21	0,008	0,007	0,006	0,003	0,004	0,013	0,003	0,005	0,008	0,006	0,014	0,001	0,007	0,003	0,005	0,001	0,012	0,005	0,004	0,006	0,004	0,004	0,001	0,005
XOA60	21	0,012	0,009	0,008	0,003	0,002	0,008	0,004	0,005	0,010	0,006	0,016	0,001	0,007	0,003	0,006	0,001	0,010	0,004	0,005	0,005	0,005	0,003	0,001	0,005
XOA91	21	0,009	0,009	0,005	0,003	0,003	0,014	0,002	0,012	0,006	0,008	0,015	0,002	0,013	0,003	0,008	0,001	0,020	0,005	0,006	0,004	0,013	0,003	0,001	0,012
XOA99	21	0,007	0,005	0,006	0,003	0,001	0,004	0,003	0,004	0,008	0,007	0,007	0,001	0,004	0,003	0,004	0,004	0,006	0,004	0,003	0,005	0,004	0,003	0,001	0,003
XOA88	20	0,008	0,006	0,007	0,005	0,001	0,004	0,003	0,004	0,007	0,006	0,007	0,001	0,004	0,004	0,005	0,002	0,004	0,006	0,004	0,007	0,003	0,005	0,002	0,004
XOA137	20	0,006	0,006	0,005	0,003	0,001	0,011	0,002	0,006	0,006	0,004	0,011	0,002	0,009	0,003	0,007	0,001	0,013	0,006	0,005	0,005	0,009	0,003	0,001	0,005
XOA185	19	0,004	0,004	0,011	0,005	0,001	0,006	0,004	0,005	0,013	0,005	0,008	0,001	0,005	0,005	0,003	0,001	0,007	0,006	0,002	0,008	0,005	0,005	0,002	0,003
XOA187	19	0,005	0,005	0,006	0,002	0,002	0,006	0,003	0,007	0,007	0,020	0,011	0,001	0,006	0,002	0,004	0,002	0,007	0,006	0,003	0,005	0,005	0,002	0,001	0,007
XOA21	18	0,004	0,003	0,007	0,003	0,001	0,006	0,002	0,005	0,007	0,005	0,009	0,001	0,005	0,003	0,003	0,001	0,007	0,005	0,002	0,004	0,005	0,002	0,001	0,011
XOA31	18	0,011	0,010	0,004	0,003	0,001	0,011	0,002	0,004	0,006	0,006	0,011	0,001	0,010	0,002	0,008	0,001	0,011	0,004	0,005	0,004	0,005	0,002	0,002	0,003
XOA153	18	0,007	0,007	0,004	0,003	0,002	0,020	0,002	0,009	0,003	0,002	0,014	0,002	0,013	0,003	0,008	0,001	0,016	0,007	0,007	0,004	0,013	0,003	0,001	0,010
XOA159	18	0,006	0,006	0,006	0,002	0,002	0,006	0,006	0,010	0,008	0,005	0,014	0,002	0,005	0,002	0,004	0,017	0,006	0,007	0,003	0,005	0,005	0,002	0,001	0,007
XOA45	17	0,006	0,005	0,005	0,003	0,001	0,012	0,020	0,003	0,005	0,007	0,007	0,014	0,005	0,002	0,004	0,001	0,006	0,003	0,004	0,004	0,004	0,003	0,001	0,002
XOA61	17	0,005	0,004	0,005	0,003	0,001	0,005	0,002	0,004	0,006	0,005	0,013	0,001	0,005	0,003	0,003	0,001	0,007	0,004	0,003	0,004	0,004	0,003	0,001	0,003
XOA19	16	0,005	0,005	0,004	0,003	0,001	0,007	0,002	0,005	0,004	0,004	0,007	0,002	0,006	0,003	0,005	0,001	0,009	0,005	0,004	0,004	0,007	0,003	0,001	0,004
XOA163	16	0,011	0,010	0,007	0,002	0,001	0,004	0,002	0,004	0,009	0,005	0,010	0,001	0,005	0,002	0,006	0,001	0,005	0,004	0,004	0,005	0,004	0,003	0,001	0,003
XOA23	15	0,004	0,004	0,005	0,002	0,002	0,004	0,002	0,004	0,006	0,008	0,007	0,002	0,004	0,002	0,003	0,001	0,005	0,005	0,002	0,005	0,004	0,003	0,001	0,004
XOA106	15	0,005	0,004	0,004	0,002	0,001	0,007	0,002	0,006	0,004	0,003	0,008	0,001	0,006	0,002	0,004	0,001	0,008	0,005	0,004	0,004	0,006	0,002	0,001	0,007
XOA79	14	0,004	0,004	0,004	0,003	0,002	0,004	0,003	0,003	0,005	0,004	0,004	0,001	0,004	0,003	0,003	0,001	0,007	0,004	0,003	0,004	0,006	0,003	0,001	0,003
XOA92	14	0,005	0,004	0,005	0,003	0,006	0,005	0,002	0,009	0,007	0,004	0,013	0,001	0,005	0,002	0,003	0,001	0,006	0,003	0,002	0,008	0,007	0,003	0,001	0,003
XOA112	14	0,006	0,005	0,005	0,004	0,001	0,004	0,003	0,003	0,006	0,004	0,005	0,001	0,003	0,003	0,005	0,001	0,004	0,006	0,004	0,005	0,003	0,004	0,001	0,004
XOA125	14	0,004	0,004	0,004	0,003	0,001	0,005	0,003	0,006	0,005	0,003	0,007	0,001	0,004	0,002	0,003	0,003	0,005	0,006	0,002	0,003	0,004	0,002	0,001	0,012
XOA89	13	0,004	0,004	0,004	0,004	0,002	0,005	0,003	0,004	0,005	0,004	0,006	0,001	0,004	0,003	0,003	0,002	0,005	0,003	0,003	0,004	0,003	0,004	0,001	0,007
XOA73	12	0,005	0,004	0,004	0,003	0,001	0,004	0,002	0,004	0,005	0,003	0,006	0,001	0,004	0,002	0,004	0,001	0,005	0,004	0,003	0,004	0,004	0,003	0,001	0,003
XOA182	12	0,003	0,002	0,003	0,003	0,001	0,006	0,002	0,003	0,004	0,004	0,005	0,001	0,005	0,003	0,003	0,001	0,007	0,003	0,003	0,003	0,005	0,004	0,001	0,017
XOA33	11	0,004	0,004	0,004	0,002	0,001	0,005	0,002	0,004	0,005	0,003	0,006	0,001	0,004	0,002	0,003	0,001	0,006	0,004	0,003	0,004	0,004	0,003	0,001	0,003
XOA145	11	0,003	0,003	0,004	0,003	0,001	0,006	0,002	0,003	0,004	0,018	0,006	0,001	0,005	0,002	0,003	0,001	0,013	0,004	0,003	0,003	0,004	0,003	0,001	0,003
XOA13	10	0,004	0,003	0,004	0,002	0,001	0,005	0,003	0,003	0,005	0,004	0,006	0,001	0,004	0,003	0,003	0,001	0,005	0,004	0,003	0,003	0,004	0,003	0,001	0,002
XOA69	10	0,003	0,002	0,006	0,001	0,001	0,006	0,002	0,004	0,006	0,004	0,009	0,001	0,006	0,001	0,002	0,001	0,006	0,004	0,001	0,003	0,005	0,002	0,001	0,005
XOA126	10	0,006	0,005	0,004	0,002	0,001	0,004	0,002	0,004	0,005	0,003	0,008	0,001	0,004	0,002	0,003	0,001	0,005	0,003	0,002	0,004	0,004	0,002	0,001	0,004
XOA138	10	0,003	0,004	0,005	0,003	0,001	0,003	0,002	0,003	0,005	0,004	0,005	0,001	0,003	0,002	0,003	0,001	0,004	0,004	0,002	0,004	0,003	0,004	0,006	0,003
XOA168	10	0,012	0,008	0,006	0,003	0,001	0,002	0,002	0,002	0,007	0,005	0,005	0,001	0,002	0,003	0,005	0,000	0,001	0,004	0,003	0,005	0,002	0,003	0,001	0,004
XOA104	9	0,005	0,004	0,003	0,003	0,001	0,003	0,003	0,002	0,003	0,004	0,003	0,002	0,002	0,003	0,004	0,001	0,003	0,002	0,004	0,003	0,002	0,003	0,001	0,004
XOA170	9	0,003	0,003	0,003	0,002	0,001	0,006	0,002	0,004	0,003	0,002	0,006	0,001	0,005	0,002	0,003	0,001	0,007	0,004	0,002	0,003	0,005	0,002	0,001	0,004
XOA72	8	0,007	0,007	0,003	0,004	0,001	0,004	0,002	0,003	0,004	0,003	0,005	0,011	0,005	0,002	0,006	0,001	0,004	0,004	0,005	0,003	0,003	0,002	0,001	0,004
XOA93	8	0,004	0,004	0,004	0,002	0,001	0,003	0,002	0,004	0,005	0,003	0,012	0,001	0,005	0,001	0,003	0,001	0,004	0,003	0,002	0,003	0,003	0,002	0,001	0,003
XOA16	7	0,006	0,004	0,004	0,002	0,001	0,002	0,003	0,002	0,004	0,004	0,004	0,001	0,002	0,003	0,003	0,001	0,002	0,005	0,002	0,004	0,002	0,003	0,001	0,003
XOA37	7	0,006	0,004	0,004	0,001	0,001	0,003	0,002	0,004	0,005	0,002	0,010	0,001	0,004	0,001	0,003	0,001	0,003	0,001	0,001	0,003	0,003	0,001	0,000	0,005
XOA46	7	0,006	0,005	0,005	0,002	0,001	0,003	0,002	0,003	0,005	0,005	0,004	0,001	0,002	0,002	0,004	0,000	0,002	0,004	0,003	0,004	0,002	0,003	0,001	0,002
XOA87	7	0,003	0,003	0,005	0,003	0,001	0,015	0,003	0,004	0,005	0,003	0,006	0,001	0,004	0,002	0,002	0,001	0,005	0,004	0,002	0,004	0,004	0,003	0,001	0,007
XOA129	7	0,004	0,004	0,004	0,002	0,001	0,004	0,002	0,003	0,004	0,003	0,005	0,001	0,015	0,002	0,006	0,001	0,005	0,003	0,003	0,003	0,003	0,002	0,001	0,003
XOA134	7	0,004	0,003	0,003	0,003	0,001	0,004	0,002	0,003	0,003	0,005	0,005	0,001	0,004	0,002	0,004	0,000	0,005	0,003	0,003	0,003	0,004	0,003	0,001	0,002
XOA135	7	0,004	0,003	0,004	0,003	0,001	0,004	0,002	0,003	0,004	0,004	0,005	0,004	0,004	0,002	0,003	0,000	0,005	0,003	0,003	0,003	0,004	0,002	0,001	0,003
XOA52	6	0,003	0,003	0,004	0,002	0,001	0,005	0,002	0,004	0,004	0,003	0,006	0,001	0,006	0,002	0,003	0,001	0,006	0,003	0,002	0,003	0,004	0,002	0,001	0,003
XOA96	6	0,005	0,004	0,004	0,003	0,001	0,002	0,003	0,002	0,005	0,003	0,004	0,001	0,002	0,002	0,003	0,001	0,002	0,003	0,002	0,004	0,002	0,003	0,001	0,003
XOA123	6	0,003	0,003	0,003	0,003	0,001	0,005	0,003	0,004	0,004	0,002	0,005	0,001	0,004	0,003	0,003	0,001	0,005	0,004	0,002	0,003	0,004	0,003	0,001	0,003
XOA120	5	0,004	0,003	0,003	0,003	0,001	0,002	0,002	0,002	0,003	0,002	0,003	0,001	0,002	0,003	0,004	0,001	0,003	0,003	0,003	0,003	0,002	0,003	0,001	0,002
XOA132	5	0,004	0,003	0,003	0,002	0,001	0,019	0,002	0,003	0,003	0,003	0,005	0,001	0,003	0,002	0,003	0,000	0,004	0,003	0,003	0,004	0,003	0,002	0,001	0,002
XOA24	4	0,005	0,003	0,003	0,002	0,001	0,002	0,001	0,002	0,003	0,020	0,003	0,000	0,002	0,003	0,002	0,000	0,001	0,002	0,002	0,003	0,001	0,002	0,001	0,003
XOA65	4	0,003	0,002	0,003	0,002	0,001	0,003	0,003	0,003	0,004	0,003	0,005	0,001	0,003	0,001	0,002	0,001	0,004	0,004	0,002	0,003	0,004	0,002	0,001	0,006
XOA85	4	0,004	0,004	0,003	0,002	0,001	0,010	0,001	0,003	0,003	0,013	0,005	0,001	0,004	0,001	0,003	0,001	0,005	0,002	0,002	0,002	0,003	0,002	0,001	0,003
XOA86	4	0,003	0,003	0,003	0,002	0,002	0,004	0,002	0,004	0,003	0,002	0,005	0,001	0,004	0,001	0,002	0,012	0,004	0,003	0,002	0,003	0,004	0,002	0,001	0,002
XOA152	4	0,010	0,006	0,005	0,001	0,001	0,001	0,001	0,002	0,005	0,003	0,005	0,000	0,002	0,001	0,003	0,000	0,001	0,003	0,002	0,004	0,001	0,002	0,001	0,002
XOA177	4	0,004	0,003	0,003	0,002	0,001	0,003	0,001	0,003	0,004	0,004	0,005	0,001	0,003	0,002	0,002	0,000	0,004	0,002	0,003	0,003	0,003	0,002	0,001	0,003
XOA42	3	0,003	0,003	0,003	0,002	0,001	0,004	0,002	0,003	0,003	0,002	0,005	0,001	0,004	0,002	0,003	0,001	0,005	0,002	0,003	0,002	0,003	0,002	0,001	0,002
XOA54	3	0,003	0,003	0,002	0,001	0,001	0,004	0,001	0,003	0,003	0,019	0,005	0,001	0,004	0,001	0,002	0,001	0,010	0,003	0,001	0,002	0,003	0,001	0,001	0,002
XOA64	3	0,006	0,004	0,003	0,002	0,001	0,002	0,002	0,002	0,003	0,003	0,004	0,001	0,002	0,002	0,003	0,002	0,002	0,002	0,002	0,003	0,002	0,003	0,001	0,002
XOA162	3	0,004	0,003	0,003	0,002	0,001	0,004	0,002	0,003	0,003	0,005	0,004	0,001	0,003	0,002	0,003	0,001	0,005	0,003	0,003	0,003	0,003	0,002	0,001	0,002
XOA70	2	0,003	0,003	0,003	0,002	0,001	0,004	0,002	0,003	0,004	0,002	0,005	0,001	0,003	0,002	0,002	0,001	0,004	0,003	0,002	0,003	0,003	0,002	0,001	0,003
XOA78	2	0,003	0,002	0,003	0,002	0,001	0,003	0,001	0,003	0,003	0,011	0,005	0,001	0,003	0,002	0,002	0,001	0,003	0,002	0,002	0,002	0,002	0,002	0,001	0,007
XOA82	2	0,003	0,003	0,002	0,001	0,001	0,007	0,001	0,003	0,003	0,002	0,005	0,001	0,003	0,001	0,002	0,000	0,004	0,002	0,002	0,002	0,003	0,001	0,001	0,003
XOA90	2	0,003	0,004	0,002	0,001	0,001	0,004	0,001	0,003	0,003	0,002	0,005	0,001	0,003	0,002	0,003	0,000	0,003	0,002	0,002	0,002	0,003	0,002	0,000	0,003
XOA121	2	0,003	0,003	0,003	0,002	0,001	0,004	0,001	0,003	0,003	0,002	0,005	0,001	0,004	0,002	0,003	0,001	0,004	0,002	0,002	0,003	0,004	0,002	0,001	0,002
XOA136	2	0,004	0,003	0,003	0,002	0,001	0,002	0,002	0,003	0,004	0,003	0,003	0,000	0,002	0,002	0,002	0,001	0,002	0,002	0,002	0,005	0,003	0,002	0,001	0,005
XOA166	2	0,003	0,002	0,002	0,003	0,001	0,003	0,001	0,002	0,003	0,003	0,004	0,000	0,002	0,004	0,002	0,000	0,003	0,002	0,002	0,002	0,002	0,002	0,001	0,002
XOA1	1	0,002	0,002	0,002	0,002	0,001	0,006	0,002	0,002	0,002	0,002	0,004	0,001	0,003	0,002	0,002	0,001	0,004	0,002	0,002	0,002	0,003	0,002	0,001	0,002
XOA14	1	0,003	0,002	0,003	0,002	0,001	0,003	0,003	0,002	0,003	0,002	0,003	0,001	0,003	0,002	0,002	0,000	0,003	0,002	0,002	0,002	0,003	0,002	0,001	0,007
XOA34	1	0,002	0,001	0,002	0,001	0,000	0,002	0,001	0,002	0,002	0,002	0,004	0,001	0,002	0,001	0,001	0,000	0,003	0,003	0,001	0,002	0,002	0,001	0,001	0,002
XOA53	1	0,001	0,001	0,003	0,002	0,001	0,003	0,002	0,002	0,003	0,002	0,004	0,000	0,003	0,002	0,001	0,000	0,004	0,003	0,001	0,003	0,003	0,002	0,001	0,003
XOA55	1	0,003	0,002	0,003	0,002	0,001	0,004	0,002	0,003	0,003	0,006	0,005	0,001	0,003	0,002	0,002	0,001	0,005	0,003	0,002	0,003	0,004	0,002	0,001	0,004
XOA76	1	0,003	0,003	0,001	0,001	0,001	0,003	0,001	0,002	0,002	0,002	0,004	0,001	0,003	0,001	0,002	0,001	0,004	0,001	0,002	0,001	0,003	0,001	0,001	0,002
XOA111	1	0,001	0,001	0,001	0,001	0,000	0,002	0,001	0,001	0,001	0,001	0,003	0,000	0,002	0,001	0,001	0,000	0,003	0,001	0,001	0,001	0,002	0,001	0,000	0,020
XOA117	1	0,003	0,003	0,002	0,001	0,001	0,003	0,001	0,002	0,003	0,002	0,004	0,000	0,003	0,001	0,002	0,000	0,003	0,002	0,002	0,002	0,003	0,002	0,001	0,002
XOA116	1	0,003	0,003	0,002	0,001	0,000	0,002	0,001	0,002	0,002	0,001	0,003	0,000	0,002	0,001	0,003	0,000	0,003	0,001	0,002	0,002	0,002	0,001	0,001	0,001
XOA148	1	0,004	0,003	0,003	0,002	0,001	0,003	0,002	0,002	0,003	0,002	0,004	0,001	0,003	0,002	0,002	0,001	0,003	0,003	0,002	0,002	0,003	0,002	0,001	0,002
XOA155	1	0,002	0,001	0,001	0,001	0,001	0,003	0,001	0,003	0,001	0,002	0,003	0,000	0,003	0,001	0,002	0,002	0,004	0,002	0,001	0,001	0,003	0,001	0,001	0,001
XOA161	1	0,003	0,002	0,002	0,001	0,001	0,003	0,001	0,002	0,002	0,001	0,004	0,001	0,003	0,001	0,005	0,001	0,003	0,002	0,002	0,002	0,003	0,002	0,001	0,002
XOA178	1	0,002	0,001	0,001	0,001	0,003	0,002	0,001	0,002	0,001	0,001	0,003	0,000	0,002	0,001	0,002	0,000	0,003	0,001	0,001	0,001	0,003	0,001	0,000	0,002
XOA184	1	0,003	0,002	0,002	0,001	0,001	0,002	0,001	0,001	0,002	0,002	0,002	0,000	0,001	0,001	0,002	0,001	0,001	0,002	0,002	0,002	0,001	0,001	0,001	0,009
XOA3	0	0,002	0,001	0,001	0,001	0,000	0,002	0,001	0,002	0,002	0,002	0,003	0,000	0,003	0,001	0,002	0,000	0,003	0,002	0,002	0,001	0,002	0,001	0,001	0,004
XOA5	0	0,002	0,001	0,001	0,001	0,000	0,002	0,001	0,002	0,002	0,001	0,003	0,000	0,002	0,001	0,001	0,000	0,002	0,001	0,001	0,001	0,002	0,001	0,001	0,005
XOA7	0	0,003	0,003	0,003	0,002	0,001	0,004	0,002	0,003	0,003	0,002	0,005	0,001	0,004	0,002	0,003	0,001	0,005	0,003	0,003	0,003	0,004	0,002	0,001	0,003
XOA9	0	0,002	0,002	0,002	0,001	0,001	0,003	0,001	0,002	0,004	0,003	0,003	0,000	0,003	0,001	0,002	0,000	0,003	0,002	0,002	0,002	0,003	0,002	0,001	0,006
XOA11	0	0,003	0,002	0,003	0,002	0,001	0,003	0,002	0,002	0,003	0,002	0,004	0,001	0,003	0,002	0,003	0,000	0,004	0,002	0,003	0,002	0,003	0,002	0,001	0,005
XOA20	0	0,002	0,002	0,002	0,001	0,000	0,003	0,001	0,002	0,002	0,001	0,003	0,000	0,003	0,001	0,001	0,000	0,004	0,002	0,002	0,002	0,003	0,001	0,001	0,003
XOA18	0	0,002	0,002	0,001	0,001	0,000	0,002	0,001	0,002	0,002	0,001	0,003	0,000	0,002	0,001	0,001	0,000	0,003	0,001	0,001	0,001	0,002	0,001	0,001	0,002
XOA57	0	0,003	0,002	0,002	0,001	0,001	0,003	0,002	0,002	0,003	0,002	0,004	0,000	0,003	0,002	0,002	0,001	0,003	0,002	0,002	0,002	0,003	0,002	0,001	0,002
XOA81	0	0,003	0,002	0,002	0,001	0,001	0,004	0,001	0,003	0,003	0,002	0,005	0,000	0,004	0,001	0,002	0,000	0,004	0,002	0,001	0,002	0,003	0,002	0,001	0,004
XOA84	0	0,002	0,002	0,002	0,001	0,000	0,002	0,001	0,002	0,003	0,003	0,004	0,000	0,002	0,001	0,001	0,000	0,003	0,002	0,001	0,002	0,002	0,001	0,001	0,002
XOA98	0	0,002	0,002	0,002	0,001	0,001	0,002	0,001	0,002	0,003	0,002	0,003	0,001	0,002	0,002	0,002	0,001	0,003	0,002	0,002	0,002	0,003	0,002	0,001	0,002
XOA102	0	0,002	0,002	0,002	0,001	0,000	0,002	0,001	0,001	0,002	0,001	0,003	0,000	0,002	0,001	0,002	0,000	0,003	0,002	0,002	0,002	0,002	0,002	0,001	0,001
XOA103	0	0,002	0,002	0,002	0,001	0,000	0,002	0,001	0,002	0,002	0,002	0,003	0,000	0,002	0,001	0,002	0,000	0,002	0,002	0,001	0,001	0,002	0,001	0,001	0,001
XOA107	0	0,002	0,002	0,002	0,001	0,001	0,003	0,001	0,002	0,002	0,003	0,004	0,001	0,003	0,001	0,002	0,000	0,004	0,002	0,002	0,002	0,003	0,001	0,001	0,002
XOA108	0	0,001	0,001	0,001	0,001	0,000	0,002	0,001	0,001	0,001	0,001	0,002	0,000	0,002	0,001	0,001	0,000	0,002	0,001	0,001	0,001	0,002	0,001	0,000	0,001
XOA114	0	0,002	0,002	0,002	0,001	0,000	0,002	0,001	0,002	0,002	0,002	0,003	0,000	0,002	0,001	0,002	0,000	0,002	0,002	0,002	0,001	0,002	0,001	0,001	0,001
XOA119	0	0,003	0,002	0,002	0,002	0,000	0,003	0,001	0,002	0,003	0,002	0,003	0,001	0,002	0,001	0,003	0,001	0,003	0,002	0,002	0,002	0,002	0,001	0,000	0,002
XOA124	0	0,001	0,001	0,002	0,001	0,001	0,001	0,001	0,002	0,002	0,001	0,003	0,000	0,002	0,001	0,001	0,001	0,002	0,002	0,001	0,002	0,002	0,001	0,001	0,002
XOA128	0	0,002	0,001	0,002	0,001	0,000	0,001	0,001	0,001	0,002	0,002	0,002	0,000	0,004	0,001	0,003	0,000	0,001	0,002	0,001	0,002	0,001	0,001	0,001	0,001
XOA139	0	0,002	0,002	0,003	0,002	0,001	0,003	0,001	0,002	0,003	0,003	0,004	0,000	0,003	0,002	0,002	0,000	0,004	0,003	0,001	0,002	0,003	0,001	0,001	0,002
XOA140	0	0,002	0,002	0,001	0,001	0,000	0,002	0,001	0,002	0,001	0,001	0,003	0,000	0,002	0,001	0,001	0,000	0,003	0,001	0,001	0,001	0,003	0,001	0,001	0,002
XOA141	0	0,001	0,001	0,001	0,001	0,000	0,002	0,001	0,001	0,001	0,001	0,003	0,000	0,002	0,001	0,001	0,000	0,002	0,001	0,001	0,001	0,002	0,001	0,000	0,001
XOA142	0	0,002	0,002	0,002	0,001	0,001	0,003	0,001	0,002	0,002	0,002	0,004	0,000	0,003	0,001	0,002	0,000	0,004	0,002	0,002	0,002	0,003	0,001	0,001	0,002
XOA143	0	0,001	0,001	0,001	0,001	0,001	0,002	0,001	0,002	0,001	0,001	0,003	0,000	0,002	0,001	0,001	0,000	0,003	0,001	0,001	0,001	0,002	0,001	0,001	0,002
XOA146	0	0,002	0,002	0,002	0,001	0,000	0,002	0,001	0,002	0,002	0,002	0,003	0,000	0,002	0,001	0,002	0,000	0,002	0,002	0,002	0,001	0,002	0,001	0,001	0,001
XOA147	0	0,003	0,002	0,002	0,001	0,000	0,002	0,001	0,002	0,002	0,002	0,003	0,001	0,002	0,001	0,003	0,000	0,003	0,002	0,002	0,002	0,002	0,002	0,001	0,002
XOA149	0	0,001	0,001	0,001	0,001	0,000	0,001	0,001	0,001	0,001	0,001	0,002	0,000	0,001	0,001	0,001	0,000	0,002	0,001	0,001	0,001	0,002	0,001	0,000	0,001
XOA150	0	0,002	0,002	0,002	0,001	0,001	0,003	0,001	0,002	0,002	0,001	0,003	0,001	0,002	0,001	0,002	0,000	0,003	0,002	0,002	0,001	0,003	0,001	0,001	0,003
XOA154	0	0,001	0,001	0,001	0,001	0,000	0,002	0,001	0,001	0,001	0,001	0,002	0,000	0,002	0,001	0,001	0,000	0,002	0,001	0,001	0,001	0,002	0,001	0,000	0,001
XOA164	0	0,003	0,002	0,002	0,002	0,001	0,002	0,001	0,002	0,002	0,002	0,003	0,001	0,002	0,001	0,002	0,000	0,003	0,002	0,002	0,002	0,002	0,002	0,001	0,001
XOA169	0	0,002	0,002	0,002	0,001	0,000	0,003	0,001	0,002	0,002	0,001	0,004	0,000	0,003	0,001	0,002	0,000	0,003	0,001	0,001	0,002	0,003	0,001	0,000	0,002
XOA175	0	0,001	0,001	0,001	0,001	0,000	0,002	0,001	0,002	0,001	0,001	0,003	0,000	0,002	0,001	0,001	0,001	0,003	0,001	0,001	0,001	0,002	0,001	0,001	0,003
XOA176	0	0,002	0,002	0,001	0,001	0,000	0,001	0,001	0,001	0,001	0,001	0,001	0,000	0,001	0,001	0,001	0,000	0,001	0,001	0,001	0,001	0,001	0,001	0,000	0,001
XOA180	0	0,002	0,001	0,002	0,001	0,001	0,002	0,001	0,002	0,002	0,002	0,003	0,000	0,002	0,001	0,002	0,000	0,003	0,001	0,001	0,002	0,002	0,001	0,001	0,006
XOA186	0	0,002	0,002	0,002	0,001	0,001	0,003	0,001	0,002	0,002	0,002	0,004	0,000	0,003	0,001	0,002	0,000	0,004	0,002	0,002	0,002	0,004	0,001	0,001	0,002
XOA189	0	0,001	0,001	0,001	0,001	0,000	0,003	0,001	0,002	0,002	0,001	0,004	0,000	0,003	0,001	0,001	0,000	0,003	0,001	0,000	0,001	0,002	0,001	0,000	0,002

S7 Fig. Reactivity pattern of sera samples against the P. vivax coiled coil fragments Light blue: The cut-off for positivity was determined as the mean+3 standard deviations of negative. Middle blue: 2X cut-off. Dark blue >3X cut off.
