# Supplementary material for: Natural immune response to Plasmodium vivax alpha-helical coiled coil protein motifs and its association with the risk of P. vivax malaria
Source: PLoS One. 2017 Jun 26;12(6):e0179863. doi: 10.1371/journal.pone.0179863 (PMC5484505; doi:10.1371/journal.pone.0179863)
Supplement: S1 Table — (DOCX) [file pone.0179863.s002.docx]

**S1 Table. Prevalence of responders to *P. vivax* coiled coil fragments in young children from Ilahita and Sunuhu villages.**

| Antigen | Ilahita^a^ | Sunuhu^b^ | p value^c^ |
| --- | --- | --- | --- |
| Pv5 | 38 (37%) | 27 (44%) | 0.388 |
| Pv12 | 45 (44%) | 37 (60%) | 0.034* |
| Pv27 | 34 (33%) | 23 (37%) | 0.657 |
| Pv40 | 49 (48%) | 35 (56%) | 0.322 |
| Pv42 | 44 (43%) | 32 (52%) | 0.257 |
| Pv43 | 41 (40%) | 31 (50%) | 0.201 |
| Pv45 | 40 (39%) | 21 (34%) | 0.557 |
| Pv52 | 47 (46%) | 36 (58%) | 0.119 |
| Pv63 | 38 (37%) | 33 (53%) | 0.033* |
| Pv81 | 39 (38%) | 27 (44%) | 0.472 |
| Pv82.02 | 60 (59%) | 45 (73%) | 0.052 |
| Pv82.03 | 48 (47%) | 39 (63%) | 0.033* |
| Pv83 | 41 (40%) | 31 (50%) | 0.201 |
| Pv90 | 20 (20%) | 11 (18%) | 0.857 |
| Pv92 | 32 (31%) | 26 (42%) | 0.142 |
| Pv95 | 47 (46%) | 39 (63%) | 0.023* |
| Pv96.01 | 51 (50%) | 41 (66%) | 0.201 |
| Pv96.03 | 46 (45%) | 31 (50%) | 0.571 |
| Pv101 | 42 (41%) | 25 (40%) | 1.000 |
| Pv106 | 41 (40%) | 37 (60%) | 0.007** |
| Pv112 | 45 (44%) | 33 (53%) | 0.258 |
| Pv121 | 60 (59%) | 46 (74%) | 0.036* |
| Pv123 | 42 (41%) | 29 (47%) | 0.476 |
| Pv145 | 43 (42%) | 38 (61%) | 0.011* |

^a^Samples from Ilahita children (n = 102)

^b^Samples from Sunuhu children (n = 62)

^c^P-value calculated by Fisher’s exact test between Ilahita and Sunuhu. P-values < 0.05 were considered statistically significant*
