# Supplementary material for: Natural immune response to Plasmodium vivax alpha-helical coiled coil protein motifs and its association with the risk of P. vivax malaria
Source: PLoS One. 2017 Jun 26;12(6):e0179863. doi: 10.1371/journal.pone.0179863 (PMC5484505; doi:10.1371/journal.pone.0179863)
Supplement: S2 Table — (DOCX) [file pone.0179863.s003.docx]

**S2 Table. Antibody reactivity at enrollment by age group**

| Antigen | <21months (n=81) | |  | >21 months (n=83) | |  | ^a^P value |
| --- | --- | --- | --- | --- | --- | --- | --- |
|  | Positive | negative |  | positive | negative |  |  |
| Pv5 | 34(42%) | 47(58%) |  | 31(37%) | 52(63%) |  | 0,632 |
| Pv27 | 26(32%) | 55(68%) |  | 31(37%) | 52(63%) |  | 0,515 |
| Pv43 | 34(42%) | 47(58%) |  | 38(46%) | 45(54%) |  | 0,640 |
| Pv52 | 38(47%) | 43(53%) |  | 45(54%) | 38(45%) |  | 0,435 |
| Pv82.03 | 42(52%) | 39(48%) |  | 45(54%) | 38(45%) |  | 0,876 |
| Pv92 | 24(30%) | 57(70%) |  | 34(41%) | 49(59%) |  | 0,144 |
| Pv96.01 | 46(57%) | 35(43%) |  | 46(55%) | 37(45%) |  | 0,876 |
| Pv106 | 41(51%) | 40(49%) |  | 37(45%) | 46(55%) |  | 0,532 |
| Pv101 | 36(44%) | 45(55%) |  | 31(37%) | 52(63%) |  | 0,427 |
| Pv145 | 39(48%) | 42(52%) |  | 42(51%) | 41(49%) |  | 0,758 |
| Pv12 | 43(53%) | 38(47%) |  | 39(47%) | 44(53%) |  | 0,532 |
| Pv40 | 44(54%) | 37(46%) |  | 40(48%) | 43(52%) |  | 0,440 |
| Pv42 | 41(51%) | 40(49%) |  | 35(42%) | 48(58%) |  | 0,348 |
| Pv45 | 31(38%) | 50(62%) |  | 30(36%) | 53(64%) |  | 0,872 |
| Pv63 | 35(43%) | 46(57%) |  | 36(43%) | 47(57%) |  | 1,000 |
| Pv90 | 17(21%) | 64(79%) |  | 14(17%) | 69(83%) |  | 0,553 |
| Pv95 | 42(52%) | 39(48%) |  | 44(53%) | 39(47%) |  | 1,000 |
| Pv123 | 36(44%) | 45(55%) |  | 35(42%) | 48(58%) |  | 0,875 |
| Pv112 | 41(51%) | 40(49%) |  | 37(44%) | 46(55%) |  | 0,532 |
| Pv83 | 37(46%) | 44(54%) |  | 35(42%) | 48(58%) |  | 0,753 |
| Pv81 | 32(40%) | 49(60%) |  | 34(41%) | 49(59%) |  | 0,875 |
| Pv82.02 | 57(70%) | 24(30%) |  | 48(58%) | 35(42%) |  | 0,106 |
| Pv96.03 | 38(47%) | 43(53%) |  | 39(47%) | 44(53%) |  | 1,000 |
| Pv121 | 57(70%) | 24(30%) |  | 49(59%) | 34(41%) |  | 0,144 |

^a^P values ≤0.05 were considered significant. P-value calculated by Fisher’s exact test
