# Supplementary material for: Natural immune response to Plasmodium vivax alpha-helical coiled coil protein motifs and its association with the risk of P. vivax malaria
Source: PLoS One. 2017 Jun 26;12(6):e0179863. doi: 10.1371/journal.pone.0179863 (PMC5484505; doi:10.1371/journal.pone.0179863)
Supplement: S6 Table — (DOCX) [file pone.0179863.s007.docx]

**S5 Table. Association between IgG levels to *P. vivax* antigens and protection against *P. falciparum* clinical disease (with parasite density >2500/µl of blood).**

| Antigen | ^a^IRR | ^b^95% CI | ^c^p-value |  | ^d^aIRR | ^b^95% CI | ^c^p-value |
| --- | --- | --- | --- | --- | --- | --- | --- |
| Pv5 | 1.208 | (0.879, 1.660) | 0.244 |  | 1.087 | (0.744, 1.586) | 0.667 |
| Pv12 | 1.126 | (0.797, 1.591) | 0.500 |  | 1.015 | (0.682, 1.510) | 0.943 |
| Pv27 | 1.101 | (0.774, 1.567) | 0.591 |  | 1.017 | (0.709, 1.459) | 0.927 |
| Pv40 | 1.192 | (0.832, 1.708) | 0.339 |  | 1.097 | (0.747, 1.611) | 0.635 |
| Pv42 | 1.152 | (0.779, 1.705) | 0.478 |  | 1.041 | (0.672, 1.611) | 0.858 |
| Pv43 | 1.159 | (0.849, 1.581) | 0.353 |  | 1.155 | (0.812, 1.644) | 0.422 |
| Pv45 | 0.955 | (0.653, 1.399) | 0.814 |  | 0.944 | (0.615, 1.449) | 0.792 |
| Pv52 | 1.285 | (0.934, 1.768) | 0.123 |  | 1.212 | (0.849, 1.730) | 0.290 |
| Pv63 | 1.218 | (0.924, 1.609) | 0.166 |  | 1.121 | (0.830, 1.514) | 0.456 |
| Pv81 | 1.033 | (0.728, 1.466) | 0.855 |  | 1.038 | (0.696, 1.548) | 0.856 |
| Pv82.02 | 1.226 | (0.858, 1.750) | 0.263 |  | 1.088 | (0.717, 1.649) | 0.693 |
| Pv82.03 | 1.093 | (0.792, 1.510) | 0.588 |  | 1.066 | (0.738, 1.540) | 0.732 |
| Pv83 | 1.125 | (0.779, 1.623) | 0.530 |  | 1.036 | (0.692, 1.549) | 0.864 |
| Pv90 | 1.038 | (0.724, 1.488) | 0.839 |  | 1.087 | (0.741, 1.594) | 0.671 |
| Pv92 | 1.183 | (0.854, 1.640) | 0.312 |  | 1.138 | (0.783, 1.655) | 0.499 |
| Pv95 | 1.267 | (0.916, 1.752) | 0.153 |  | 1.106 | (0.754, 1.623) | 0.606 |
| Pv96.01 | 1.260 | (0.916, 1.734) | 0.156 |  | 1.112 | (0.756, 1.636) | 0.590 |
| Pv96.03 | 1.097 | (0.840, 1.432) | 0.499 |  | 1.045 | (0.785, 1.390) | 0.763 |
| Pv101 | 1.112 | (0.795, 1.556) | 0.534 |  | 1.041 | (0.710, 1.528) | 0.834 |
| Pv106 | 1.289 | (0.941, 1.765) | 0.114 |  | 1.140 | (0.805, 1.614) | 0.460 |
| Pv112 | 1.323 | (0.896, 1.955) | 0.159 |  | 1.126 | (0.714, 1.775) | 0.610 |
| Pv123 | 1.104 | (0.751, 1.622) | 0.615 |  | 1.077 | (0.712, 1.631) | 0.725 |
| Pv121 | 1.267 | (0.870, 1.846) | 0.218 |  | 1.117 | (0.727, 1.716) | 0.613 |
| Pv145 | 1.304 | (0.946, 1.797) | 0.106 |  | 1.104 | (0.747, 1.633) | 0.618 |

^a^IRRs are derived from crude negative binomial GEE models.

^b^95% CI, 95% confidence interval;

^c^P-values ≤ 0.05 were considered statistically significant.

^d^aIRRs are negative binomial GEE models adjusted for age, village of residence, seasonality, and individual differences in exposure (_mol_FOB).

IRR, incidence rate ratio; aIRR, adjusted incidence rate ratio; GEE, generalized estimating equation.
