# Supplementary material for: Natural immune response to Plasmodium vivax alpha-helical coiled coil protein motifs and its association with the risk of P. vivax malaria
Source: PLoS One. 2017 Jun 26;12(6):e0179863. doi: 10.1371/journal.pone.0179863 (PMC5484505; doi:10.1371/journal.pone.0179863)
Supplement: S7 Table — (DOCX) [file pone.0179863.s008.docx]

**S6 Table. Prevalence of antibody responders to *P. vivax* Coiled coil fragments in donors from Burkina Faso**

| **Peptide** | **Protein** | **Ortholog** | **MW** | **Sequence** | **%^1^** | **Ratio^2^ (%)** | **Mean^3^ OD** |
| --- | --- | --- | --- | --- | --- | --- | --- |
| Pv2 | PVX_003585 | PFB0145c | 3171 | SITSLSTKIVNYETKIEDLEKELKMEK | 24 | 8 | 0,153 |
| Pv5 | PVX_003585 | PFB0145c | 2936 | IADIKISLEKLKYEVKDKKDCLENV | 43 | 32 | 0,263 |
| Pv40 | PVX_119385 | PFC0235w | 3634 | NETIQRMSNSLLKYEQDIETYQNEVSTLTGK | 32 | 16 | 0,163 |
| Pv42 | PVX_087730 | PF07_0014 | 3348 | NTPDYYKKITTKLQNNINNVEEYINNITNDINILKSSID | 32 | 8 | 0,103 |
| Pv43 | PVX_089660 | PFD0685c | 4583 | SVDINALNEQVKKLREELNKVTNEYDDFKNKLELLYQK | 30 | 16 | 0,180 |
| Pv45 | PVX_123385 | PF11_0207 | 4333 | KEVKVEVNEVGEEVNEVKEEVNEAKEEVIEKKEEMTE | 35 | 22 | 0,218 |
| Pv48 | PVX_091760 | PF11_0240 | 4133 | KGLEEANEKLQTVREKVQSLKAQLSTLISQYDHALY | 3 | 0 | 0,129 |
| Pv52 | PVX_123480 | PFL0770w | 3617 | VEQVKKEINQINEQININETKITHLRNKIE | 30 | 16 | 0,198 |
| Pv55 | PVX_122430 | PF13_0065 | 4074 | TSFSKYVRQLEQYFDNFDQDFLSLRQKISDILQ | 0 | 0 | 0,106 |
| Pv59 | PVX_118400 | PF14_0397 | 3233 | LEKSVKSIDENIDKYNKELNIIKQKIE | 5 | 3 | 0,125 |
| Pv81 | PVX_118160 | PF07_0086 | 4442 | NEMDETLSKLKKDINKLNEKIQKYDNYVKKKRKEID | 30 | 5 | 0,176 |
| Pv65 | PVX_003960 | PFB0460c | 3631 | NKLVRNKMANLKRRIDEINEELLEVANFFL | 19 | 0 | 0,147 |
| Pv72 | PVX_095365 | PFC0760c | 3216 | KEIQILKNQVSSLKQSIQTQNAFIQSLK | 19 | 3 | 0,080 |
| Pv77 | PVX_089365 | PF08_0048 | 2970 | KVAKYNEEISLLKQQLTYLNEKMGK | 65 | 11 | 0,166 |
| Pv87 | PVX_100940 | PFL1930w | 4696 | KYKIEVNILNEEIAKLKSQVSTYRNDIKNISSTLDFYKST | 14 | 5 | 0,144 |
| Pv90 | PVX_00072 | PFD0520c | 4164 | TRRMHSELSDGNKELKKLKKNIVQSDVLNAQLELNI | 22 | 16 | 0,143 |
| Pv119 | PVX_091760 | PF11_0240 | 3556 | DHVEELSKDIDNLEKDIDEIEKLWIFIKK | 38 | 19 | 0,123 |

^1^Corresponds the percentage of responders.

^2^Percentage of positive responders defined as those with OD values higher than the mean negative control+3SD.

^3^Percentage of individuals with OD ratio >2 defined as the mean duplicate experimental and mean negative control OD.
